# Oral manifestations in women using hormonal contraceptive methods: a systematic review

**DOI:** 10.1007/s00784-024-05573-x

**Published:** 2024-03-01

**Authors:** Marta García Rojo, Miguel Ramón Pecci Lloret, Julia Guerrero Gironés

**Affiliations:** Gerodontology and Special Care Dentistry Unit, Faculty of Medicine, IMIB-Arrixaca, Morales Meseguer Hospital, University of Murcia, Murcia, 30008 Spain

**Keywords:** Hormonal contraceptives, Oral manifestations, Periodontal disease, Alveolar osteitis, Oral candidiasis, Salivary microbiome

## Abstract

**Objectives:**

To investigate the oral manifestations in women of reproductive age using hormonal contraceptive methods.

**Materials and methods:**

This review is based on the PRISMA statement. A literature search incorporated observational studies from the last 21 years. An investigative question was formulated using the PICO model, studies were selected, and a quality analysis was performed using the modified STROBE guidelines. A bibliometric analysis was performed, and the data were examined.

**Results:**

Thirteen articles were included, with the majority evaluating periodontal status. Others analyzed factors such as the presence of alveolar osteitis, oral candidiasis, and salivary microbiome dysbiosis. Ten articles were deemed to have a low risk of bias.

**Conclusions:**

Hormonal contraceptives may increase the risk of alveolar osteitis following tooth extraction and increase the presence of the Candida species in the oral cavity. They also affect the periodontium, such as the frequent development of gingivitis, but do not lead to changes in the salivary microbiome.

**Clinical relevance:**

The increasing number of women using hormonal contraceptives and the knowledge that these contraceptives can produce oral cavity alterations underscore the need to evaluate the oral manifestations found in these women.

## Introduction

Throughout her life, a woman experiences hormonal fluctuations that can lead to bodily changes, having specific implications for her oral microflora [1, 2].

Hormonal contraceptives are based on synthetic combinations of estrogen and progesterone or progesterone alone, mimicking a state of pregnancy to prevent ovulation [3–5]. The mechanisms they use to hinder implantation are based on ovulation inhibition, alterations in the cervical mucus, and modifications in the endometrium [6, 7].

Progestin, which simulates endogenous progesterone and provides contraceptive action, is most commonly used [8, 9]. However, contraceptives that combine with estrogen, which participate in ovulation inhibition, regulate bleeding, and maintain endometrial thinning produced by progestin [8, 10] are also used.

In recent years, an increasing number of contraceptive methods have been developed, allowing women to choose the most suitable method based on their individual situation, needs, and preferences [11]. Additionally, these methods empower women to exert greater control over family planning, which is one of the most significant tasks of the special research program on Human Reproduction produced by the World Health Organization (WHO) [11, 12].

Combined oral contraceptives are among the most prescribed drugs worldwide [13–15]. Over time, they have undergone modifications to reduce their side effects [6, 8]. The first modification involved reducing the estrogenic component, and the second involved the development of new progestins to increase safety and limit androgenic side effects [6, 16]. The traditional way of administering oral contraceptives is in a 28-day cycle, but products with extended and continuous regimens have been introduced to reduce menstrual symptoms and the number of bleeding days. Furthermore, the hormone content in each pill can vary, with monophasic pills maintaining the exact dosage throughout the cycle, biphasic pills changing once, and triphasic pills changing twice [8, 17].

The suitable option for women with medical issues or side effects related to estrogens is the progestin-only pill, also known as the “Mini-Pill” [8, 16]. Progestin doses are low and result mainly in endometrial thinning, increased cervical mucus thickness, and reduced tubal motility. The dosage is continuous, without a hormone-free interval, and, to achieve good results, the pill must be taken every day [8, 18].

As an alternative, hormonal intrauterine devices (IUDs), which did not gain acceptance as a contraceptive method until the creation of Mirena® in 2001 [19] have become popular. This device has a T shape containing a reservoir with 52 mg of levonorgestrel and polydimethylsiloxane, which regulates its release [6]. As this method prevents fertilization, it is not considered an abortive method and is effective for at least 5 years [8].

New administration systems have recently been incorporated to improve tolerability, convenience, and compliance. These include injectable, transdermal, vaginal, and implantable systems [6, 20]. Emergency contraception, commonly known as the “morning-after pill,” contains 1.5 mg of levonorgestrel [16, 21] and is effective up to 72 h but should be taken as soon as possible after sexual intercourse. Its efficacy results from ovulation inhibition or delay, and its safety allows it to be sold without a prescription in many European countries [6, 16].

The use of hormonal contraceptives could lead to problems in the oral cavity, necessitating specific attention and care [22, 23]. The medical records obtained during a dental consultation should include the use of contraceptives, and women of reproductive age should be questioned about their use [24, 25]. The dentist must have up-to-date knowledge of these methods to properly advise patients and address any concerns that may arise [26, 27].

The increasing number of women using hormonal contraceptives and the awareness that these contraceptives can cause alterations in the oral cavity highlight the need for a systematic review synthesizing the oral manifestations that may occur in these women. Therefore, this study aimed to conduct a qualitative synthesis of studies to determine possible oral manifestations that may appear in women of reproductive age using hormonal contraceptive methods.

## Materials and methods

This systematic review was conducted following the PRISMA 2020 guidelines (Preferred Reporting Items for Systematic Reviews and Meta-Analyses) [28] and was registered with PROSPERO (International prospective register of systematic reviews) under the identification number CRD42022378210 [29]. Additionally, the PICO model [30] was used to formulate the following research question: What are the oral manifestations present in women of reproductive age using hormonal contraceptives? (P: women of reproductive age using hormonal contraceptives; I: presence of oral manifestations; C: risk of oral manifestations in women of reproductive age using hormonal contraceptives compared with those not using hormonal contraception methods; and O: prevalence of oral manifestations in women of reproductive age using hormonal contraception methods).

The search strategy, study selection process, data extraction, and quality assessment (risk of bias assessment) were performed by two independent investigators (M.G.R. and J.G.G.). In case of doubt, a third investigator was consulted (MR.P.LL.).

### Search strategy

The search was conducted in November 2023 across five electronic databases (MEDLINE, Web of Science, Scopus, Cochrane Library, and SciELO). In all databases, the search was limited to articles published between January 2002 and November 2023. The search strategy was established using the terms shown in Table 1, combined using the Boolean operators “OR” and “AND.” Additionally, advanced search symbols such as (*) were used for word truncation.

**Table 1:** Search strategy

Field 1	(contraceptives OR contraception OR “oral contraceptives” OR “hormonal contraception” OR “hormonal contraceptive” OR “birth control implant” OR “contraceptive implant” OR “implantable contraceptives” OR “hormonal iud” OR iud OR “injectable birth control” OR “injectable contraceptive” OR “injectable contraception” OR “birth control pills” OR “contraceptive pills” OR “vaginal rings” OR “contraceptive ring” OR “birth control patch” OR “contraceptive patch” OR “emergency contraception”)	
and		
Field 2	(“oral health*” OR “oral manifestation*” OR “periodontal health*” OR “periodontal disease” OR “oral disease” OR "oral cavity”)	

### Inclusion and exclusion criteria

The inclusion and exclusion criteria are presented in Table 2 and were established based on the research question and study objectives.

**Table 2:** Inclusion and exclusion criteria

Inclusion criteria	Exclusion criteria
Articles that studied oral alterations and included women of reproductive age using hormonal contraceptives.	Articles that did not include women of reproductive age using contraceptives or articles that did not study oral alterations in women of reproductive age using contraceptives.
Articles in English or Spanish	Articles in a language other than English or Spanish
Observational studies (case-control, cohort, cross-sectional, longitudinal)	Clinical cases, systematic reviews, and meta-analyses
*Studies conducted in humans*	In vitro or animal studies
Articles published within the last 21 years.	Articles published more than 21 years ago.
Articles studying oral manifestations diagnosed by medical professionals.	Articles studying oral manifestations not diagnosed by medical professionals.

### Study selection

The bibliographic references obtained through the search strategy were exported to the citation manager EndNote (Clarivate Analytics, London, United Kingdom) to remove potential duplicates. A screening process was performed by reviewing the titles and, subsequently, the abstracts based on the inclusion and exclusion criteria. Next, the articles that met these criteria were assessed for eligibility and qualitative synthesis through a full-text screening.

### Study data

For the bibliometric analysis, the following information was recorded for each article: author and year of publication, journal, and country of publication. Additionally, a table was created to summarize the following data: author and year, study design, study groups or sample, age of participants, type of hormonal contraceptive used, oral manifestations, outcomes of interest, and conclusions.

### Quality analysis

To assess the risk of bias in the selected articles, a modified STROBE (Strengthening the Reporting of Observational Studies in Epidemiology) analysis was used [31] (Table 3). This analysis consisted of 11 criteria derived from items 5, 6, 7, 8, 10, 12, 14, and 15 of the original STROBE checklist. During the evaluation, compliance with each criterion was indicated with a check mark (✓), while non-compliance was marked with a cross (×). Articles were categorized based on their scores: those with 8 to 11 points were considered as having a low risk, those with 4 to 7 points were considered as having a moderate risk, and those with 3 points or below were considered as having a high risk of bias.

**Table 3:** List of criteria used to evaluate the quality of observational studies based on an adapted version of the STROBE guidelines

Methods		
Configuration	1	Describe the environment, locations, and relevant dates, including the periods of recruitment, exposure, follow-up, and data collection.
Participants	2	Specify the eligibility criteria (inclusion and exclusion), including matched groups or control if applicable.
	3	Provide the history of hormonal contraception.
Variables	4	Clearly define the oral manifestation and its diagnostic criteria.
Data Sources / Measurement	5	Provide a detailed explanation of the evaluation methods (measurement) of the oral manifestation.
Study Size	6	Explain how the study size was determined.
Statistical Methods	7	Describe all statistical methods, including those used to control confounding factors.
	8	Describe any method used to examine subgroups and interactions.
Descriptive Data	9	Provide the characteristics of the study participants (e.g., demographic, clinical, social), and report on exposures and potential confounding factors.
	10	Indicate the number of participants with missing data and explain how it was addressed.
Outcome Data	11	Report the numbers in each exposure category or summary measures of exposure.

## Results

### Study selection and flow diagram

The literature search yielded a total of 573 results. Specifically, 96 articles were retrieved from MEDLINE (PubMed), 290 from Web of Science, 140 from Scopus, 46 from Cochrane Library, and 1 from SciELO. The results obtained from each database are summarized in Table 4.

**Table 4:** Results obtained from each database

Database	**Search strategies**	Results
MEDLINE	#1	
	#2	78,441
	#1 AND #2	98,310
Web of Science	#1	96
	#2	14,81,89,28,48,41,290
	#1 AND #2	80,674
Scopus	#1	1,15,594
	#2	140
	#1 AND #2	18,560
Cochrane Library	#1	11,600
	#2	46
	#1 AND #2	1,392
**SciELO**	#1	1,950
	#2	1
	#1 AND #2	

**Fig. 1:**
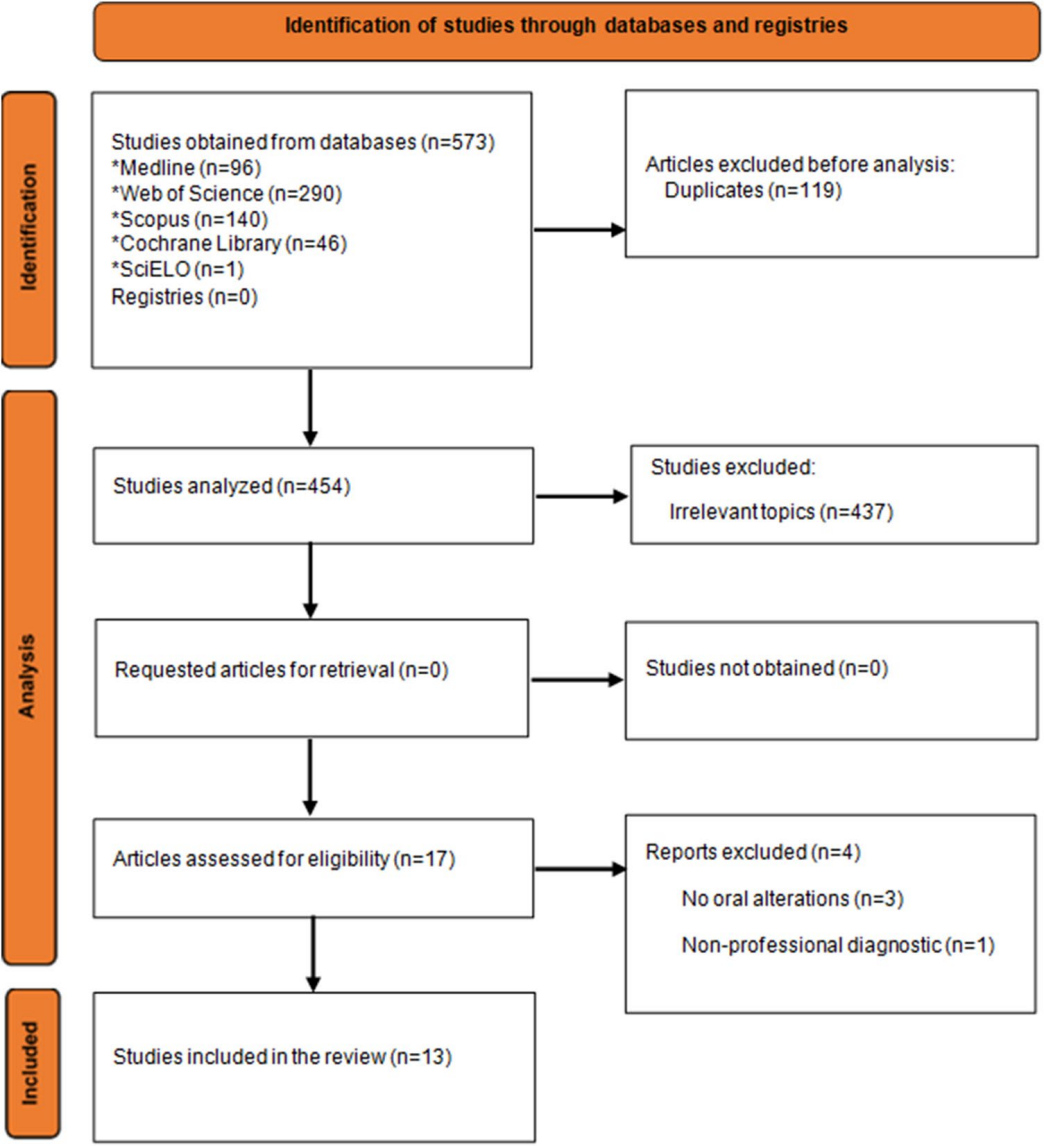
Flow diagram based on the PRISMA 2020 statement representing the study selection process for this systematic review

After discarding 119 duplicate articles, 454 were selected for title and abstract screening. Expressly, 399 articles were excluded after reviewing their titles, and an additional 55 articles were excluded after reading the abstracts and verifying that they did not meet the inclusion criteria. Next, the 17 remaining articles were assessed through full-text reading, and, ultimately, 13 articles were chosen for qualitative analysis (Fig. 1).

### Characteristics of the studies

#### Bibliometric analysis

**Fig. 2:**
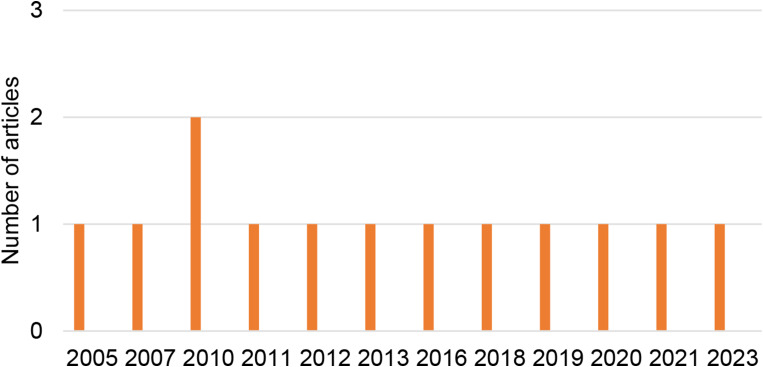
Organization of articles by year of publication

**Fig. 3:**
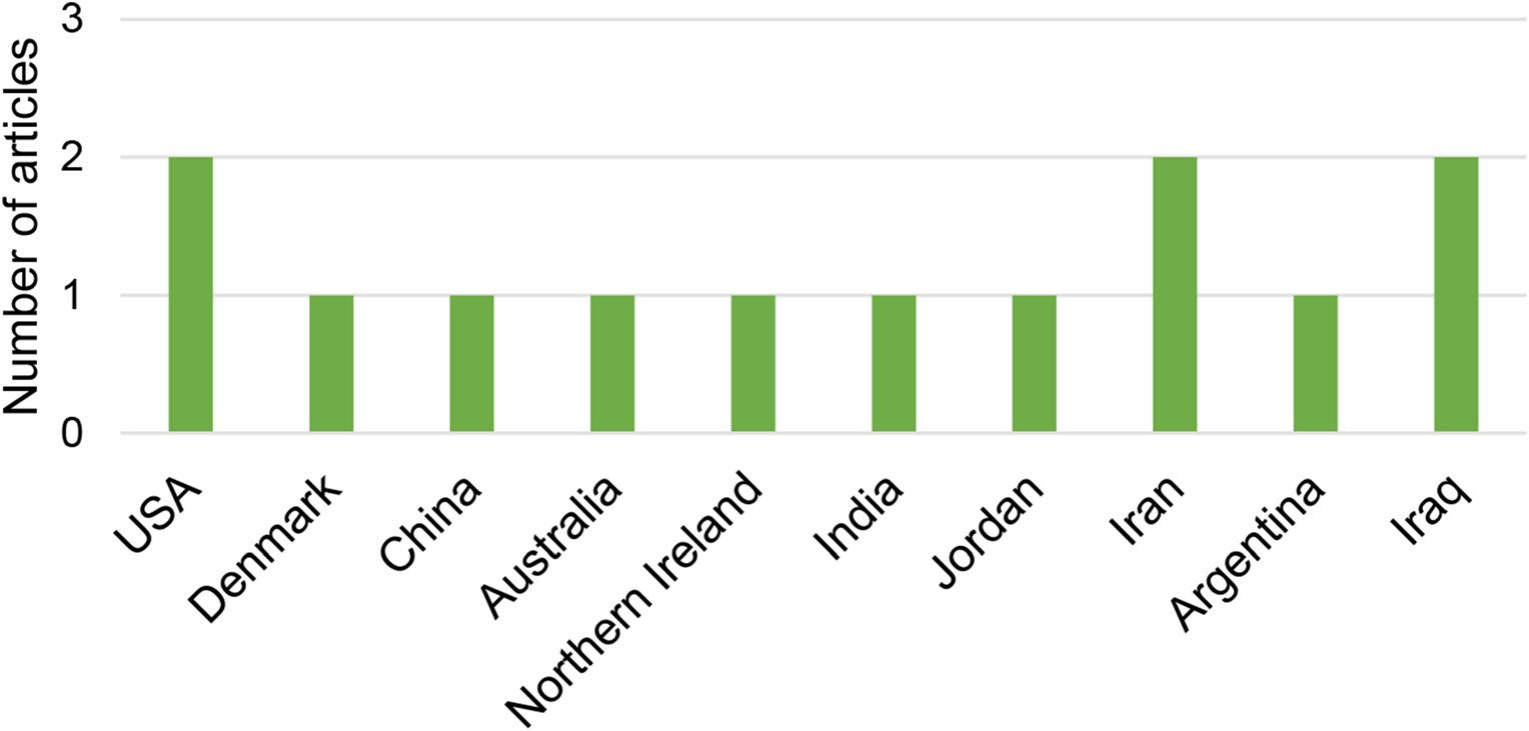
Organization of articles by country of publication

**Fig. 4:**
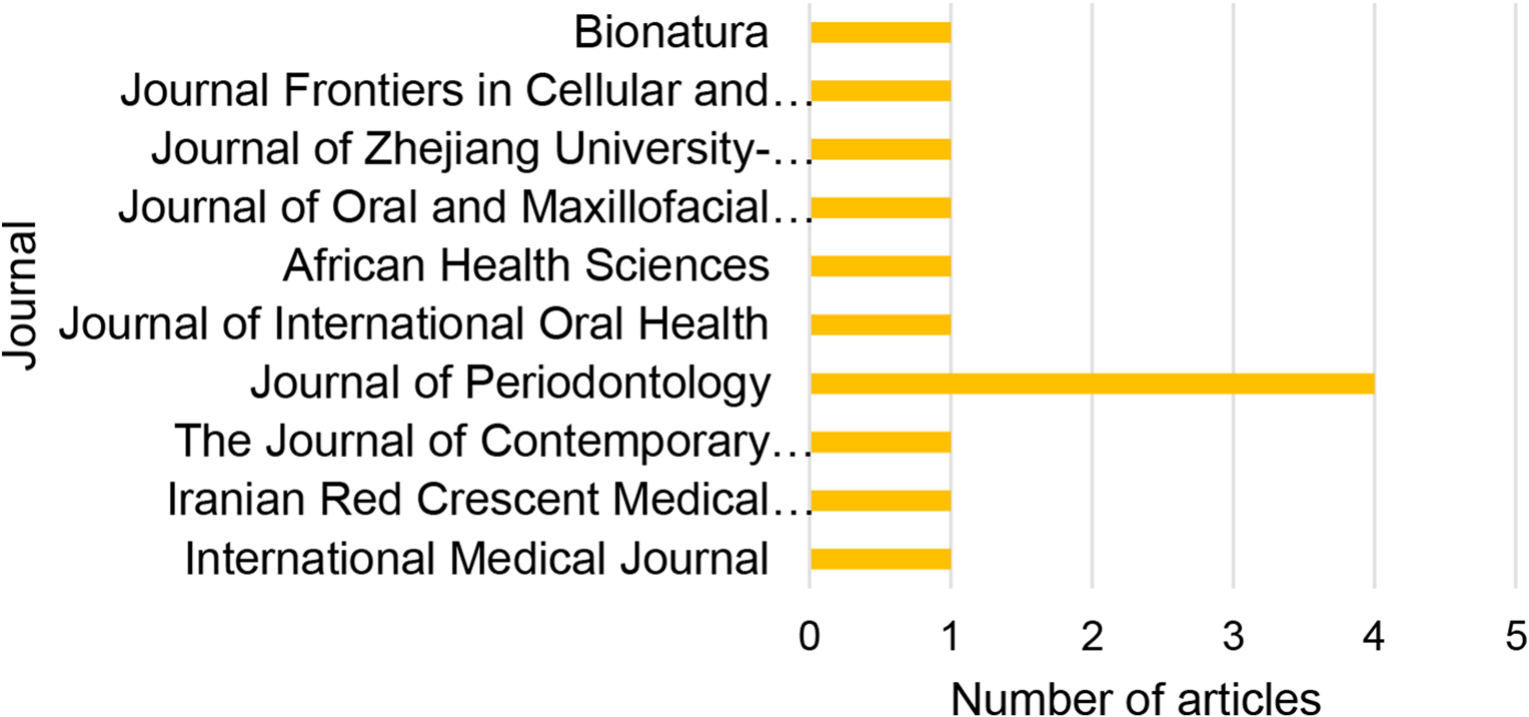
Organization of articles by journal of publication

The organization of the selected articles based on the year of publication is presented in Fig. 2, country in Fig. 3, and journal in Fig. 4.

#### Study design

Among the selected studies for the review, the following study designs were found: 6 case-control studies [32–37], which represent 46.15% of the 13 chosen articles; 5 cross-sectional studies [38–42], also accounting for 38.46% of the total; and 1 cohort study [43] and 1 longitudinal study [44], each representing 7.69% of the total (Table 5).

#### Groups or sample

The sample size was quite variable among the articles (Table 5), with sample sizes below 100 participants [32, 33, 35–37, 42], while others exceeded this value [34, 38–41, 43, 44]. Additionally, two studies [38, 44] did not specify the exact number of women using contraceptives in the total sample. It is worth noting the presence of two articles [40, 41] with sample sizes of *n* = 9931 and *n* = 4460, respectively. These articles achieved representative population samples by using data from individuals surveyed in previous years.

#### Age of participants

To indicate the age of the participants, 8 articles provided the mean age of the studied groups, representing 61.53% of the total [32, 33, 36, 37, 39, 40, 42, 43] as shown in Table 5. However, 4 articles provided an age range accounting for 30.76% [34, 35, 38, 41] of the total. One study [44] representing 7.69% of the selected articles included women using a contraceptive method but did not provide any age data,.

#### Type of hormonal contraceptive

The majority of studies include patients using oral contraceptives [32–40, 42–44], accounting for 92.30% of all selected articles. Within this group, 7 articles [32–35, 40, 42, 43] specified that the included oral contraceptives were based on combined methods. Despite including a group of women using oral contraceptives, one study [43] also evaluated another set of women using the hormonal levonorgestrel intrauterine device (IUD) as a contraceptive method. One article [41] exclusively studied a group of women using a non-oral contraceptive method, the injectable medroxyprogesterone acetate contraceptive (Table 5).

#### Oral manifestations

Among the oral manifestations listed in Table 5, those related to the periodontal status of the patients were the most prevalent and were studied in 10 articles [32–34, 36–42], accounting for 76.92% of the selected articles. Within this group, one of the articles also studied the subgingival presence of specific periodontopathogens [32], and others [36] explored various oral manifestations, such as reduced orthodontic tooth movement, ulcerative lesions, changes in mucosal color, and the presence of pyogenic granuloma. Another article [37] also studies the presence of gingival inflammation, changes in mucosal color, pyogenic granuloma and oral ulcers, but additionally measures changes in salivary flow, pH, and biochemical data such as total salivary protein, alkaline phosphatase, and immunoglobulin A (Ig A). However, some articles investigated other types of oral alterations, such as oral candidiasis [35], dysbiosis in the salivary microbiome [43], or alveolar osteitis after extraction [44].

**Table 5:** Results: main characteristics of the studies included

Author and year	Study design	Sample or population	Age of participants/(years)	Type of HC	Oral manifestations	Key outcomes of interest		Conclusions
Taichman and Eklund, 2005 [40]	cross-sectional	*n* = 9931 NHANES I = 4930 NHANES III = 5001	17–50/ NHANES I = 30 NHANES III = 32	NHANES I = High-dose COC NHANES III = Low-dose COC	Periodontal disease	NHANES I: Protective association between OC users and gingivitis (NS; OR = 0.65). OC users have a lower probability of periodontitis (OR = 0.36). NHANES III: OC users have a lower prevalence of gingivitis (OR = 80). There is no protective effect between OC users and periodontitis (OR = 0.73).		The relationship between high-dose OC and gingivitis/periodontitis is ruled out. There is an association between low-dose OC and periodontal disease non-harmful. It is premature to determine a protective effect of OC.
Mullally et al., 2007 [42]	cross-sectional	*n* = 50 (with OC 21, without OC 29)	20–35/ 29.7 ± 4.7	COC (14 users with 30 mg of EE, 4 with 35 mg of EE, 3 others)	Periodontal disease	95% of GAP diagnoses are made in individuals who take or had taken OC. OC users: higher PI and GI, but NS; higher and significant BOP (*p* = 0.017). OC users have greater mean PD (*p* = 0.006) and mean AL (*p* = 0.015). Users without a history of OC have significantly better periodontal health.		Common use of OC in women with aggressive periodontitis. High prevalence of OC use with GAP. OC users have worse periodontal health.
Brusca et al., 2010 [32]	case-control	*n* = 92 SG = 41 CG = 51	19–40/30	SG = COC (0.015 mg EE and 0.06 mg gestodene/ 0.03 mg EE and 3 mg drospirenone/ 0.02 mg EE and 3 mg drospirenone) CG = no OC	Periodontal disease and presence of specific subgingival periodontopathogens	Severe periodontitis significantly higher in SG (*p* < 0.01). SG has higher presence of *P. gingivalis* (82.9%), *P. intermedia* (85.4%), and *A. actinomycetemcomitans* (14.6%). More *Candida* species (95.1%) in SG with significant difference (*p* < 0.05). SG (during + 3 years) has a higher presence of *C. albicans, C. parapsilosis, C. krusei, C. tropicalis, and C. glabrata*, except *C. dubliniensis.*		OC increase severe periodontitis and the presence of specific periodontopathogens in periodontal pockets.
Haerian-Ardakani et al., 2010 [33]	case-control	*n* = 70 SG = 35 CG = 35	17–35/24	SG = COC Microgynon® (0.15 mg progestin and 0.03 mg EE) CG = without history	Periodontal disease	PI with no significant differences (*p* > 0.05). SG has significantly higher GI (*p* < 0.0001) and BOP (*p* < 0.001). PD and AL show no significant differences.		Users of low-dose OC for a minimum of 2 years have higher gingivitis and bleeding compared to the CG.
Parthasarathi et al., 2011 [44]	longitudinal	*n* = 284 [276 extractions in women; 14 (2.5%) in women with OC]	-	OC	Alveolar osteitis after extraction	Alveolar osteitis in 0 out of 14 extractions in women with OC.		No patient with OC developed alveolar osteitis.
Taichman et al., 2012 [41]	cross-sectional	n(NHANES) = 4460 [157 use DMPA, 553 used before, and 3750 never used]	15–44	DMPA	Periodontal disease	There is a significant association between gingivitis and current use of DMPA (OR = 1.7). The association between gingivitis and past use of DMPA is NS (*p* = 0.057). There is a modest association between periodontitis and DMPA use (OR = 1.49). DMPA users who are also smokers have a lower probability of periodontal disease (OR = 0.55).		The use of DMPA influences periodontal health.
Wu et al., 2013 [38]	cross-sectional	*n* = 754	20–39	OC	Periodontal disease	Women using OC: PD ≥ 4 mm (*p* = 0.316), CAL ≥ 3 mm (*p* = 0.309), both NS, and > 25% of sites with BOP (*p* = 0.015), which is statistically significant (*p* < 0.05).		The use of OC exacerbates gum inflammation.
Aminzadeh et al., 2016 [35]	case-control	*n* = 40 SG = 20 CG = 20	18–45	SG = COC CG = without history	Oral candidiasis	SG: significantly higher *C. albicans* (*p* = 0.04) and *C. krusei* (*p* = 0.03) but not *C. tropicalis* (*p* = 0.43).		OC increase the probability of growth of *C. albicans* and *C. krusei*.
Smadi and Zakaryia, 2018 [34]	case-control	*n* = 281 SG = 139 CG = 142	18–39	SG = COC (Yaz™, Marvelon™, Yasmin™, Microgynon30™, Dian™, others) CG = non-users	Periodontal disease	SG: OHI-S, SBI, CAL and GI significantly higher (*p* < 0.05).		COC increase the risk of gingival disease. This effect is potentiated by newer generations of COC.
Prachi et al., 2019 [39]	cross-sectional	*n* = 200 G1 = 100 G2 = 100	≥ 18 / G1 = 26,37 G2 = 27,08	G1 = OC users G2 = without history	Worse periodontal health	Mean CPI for G1 (2.34 ± 0.81) and G2 (1.16 ± 0.89). Mean LOA for G1 (0.28 ± 0.45) and G2 (0.19 ± 0.50). Significant differences in mean CPI and LOA (*p* = 0.00). Significant association between CPI and OC duration (*p* = 0.000) (+ duration worse health). Significant association between LOA and OC duration (*p* = 0.000).		OC users have worse periodontal and gingival health. Longer duration of OC use is associated with higher PD, AL, and bleeding.
Altaee, 2020 [36]	case-control	*n* = 30 SG = 15 CG = 15	18–45/ 30,6 ± 5,6	SG = OC users CG = without history	-Lesser orthodontic tooth movement. -Periodontal disease -Ulcerative lesion -Changes in mucosal color -Pyogenic granuloma		SG: significantly lower tooth movement (*p* < 0.05) and higher periodontal disease (*p* < 0.05), UL (20%), CCM (20%), PG (13.3%).	OC and FOA users show significantly less orthodontic tooth movement, significant increase in periodontal disease, and a higher percentage of CCM, PG and UL.
Bostanci et al., 2021 [43]	cohort	*n* = 103 G1 = 43 G2 = 41 G3 = 19	G1 = 23 G2 = 23 G3 = 24	G1 = does not use HC G2 = COC (20–35 µg of EE with progestins) G3 = LNG-IUD (Jaydess, Kyleena, and Mirena)	Salivary microbiome dysbiosis	Diversity and nº of species within the sample NS according to HC. HC has no notable effect on microbiome differentiation between samples. Microbiomes associated with periodontal health or disease show no notable differences between HC groups.		The use of HC is not responsible for significant changes in the salivary microbiome.
Rasheed and Ahmed, 2023 [37]	case-control	*n* = 51 SG = 30 CG = 21	16–45/ SG = 32,6 CG = 23,6	SG = OC CG = non-users	-Changes in salivary flow, pH, and biochemical data (TSP, ALP, IgA) - Gingival inflammation -CCM, UL, PG	SG: pH value NS (*P* > 0.05), significantly lower flow rate (*P* < 0.0001), significantly higher ALP (*P* < 0.05), significantly lower IgA (*P* < 0.0001), significantly lower TSP (*P* < 0.0001), no changes in oral mucosa, positive association GI-duration of therapy.		OC affect salivary flow rates and other parameters (TSP, ALP, IgA). After prolonged use of OC, the main symptom is gingival inflammation.

$\bar X$, mean; HC, hormonal contraceptive; n, total number of patients; NHANES, National Health and Nutrition Examination Survey; COC, combined oral contraceptives; OC, oral contraceptive; NS, not significant; OR, odds ratio; EE, ethinylestradiol; GAP, generalized aggressive periodontitis; PI, plaque index; GI, gingival index; BOP, bleeding on probing; PD, probing depth; AL, attachment loss; SG, study group; CG, control group; DMPA, depot medroxyprogesterone acetate; CAL, clinical attachment level; OHI−S, simplified oral hygiene index; SBI, sulcus bleeding index; G1, group 1; G2, group 2; CPI, community periodontal index; LOA, loss of attachment; UL, ulcerative lesion; CCM, change of color in mucosa; PG, pyogenic granuloma; FOA, fixed orthodontic appliance; G3, group 3; LNG−IUD, levonorgestrel−releasing intrauterine device; nº, number; TSP, total salivary protein; ALP, alkaline phosphatase; IgA, immunoglobulin A

### Quality analysis

The quality analysis used for this systematic review was based on a modified version of the STROBE guidelines for observational studies [31]. Ten studies were considered to have a low risk (76.92%), one study had moderate risk (7.69%), and two studies had high risk (15.38%) of bias. The studies classified as high risk presented only 3 of the evaluated criteria [36, 37]. The study classified as moderate risk [33] obtained a final score of 7. Among the studies with the lowest risk of bias, only one article [40] met all 11 criteria, while the rest met 10 [41, 43], 9 [32, 35, 44], and 8 [34, 38, 39, 42] criteria, respectively (Table 6).

**Table 6:** Results of the quality assessment conducted with an adapted version of the STROBE guidelines

	Taichman and Eklund [40].	Mullally et al. [42]	Brusca et al. [32]	Haerian-Ardakani et al. [33]	Parthasa-rathi et al. [44]	Taichman et al. [41]	Wu et al. [38]	Aminzadeh et al. [35]	Smadi and Zakaryia [34]	Prachi et al. [39]	Altaee [36]	Bostanci et al. [43]	Rasheed and Ahmed [37]		
1	✔	×	✔	×	✔	✔	×	✔	✔	×	×	✔		×	
2	✔	✔	✔	✔	✔	✔	✔	✔	✔	✔	×	✔		×	
3	✔	✔	✔	✔	✔	✔	✔	✔	✔	✔	✔	✔		✔	
4	✔	✔	✔	✔	✔	✔	✔	✔	✔	✔	×	✔		×	
5	✔	✔	✔	✔	✔	✔	✔	✔	×	✔	×	✔		×	
6	✔	×	×	×	×	✔	×	✔	×	✔	×	×		×	
7	✔	✔	✔	✔	✔	✔	✔	✔	✔	✔	✔	✔		✔	
8	✔	✔	✔	×	✔	✔	✔	✔	✔	✔	×	✔		×	
9	✔	✔	✔	✔	✔	✔	✔	×	✔	×	×	✔		×	
10	✔	×	×	×	×	×	×	×	×	×	×	✔		×	
11	✔	✔	✔	✔	✔	✔	✔	✔	✔	✔	✔	✔		✔	
Total Score	11	8	9	7	9	10	8	9	8	8	3	10		3	
Risk of bias	Low	Low	Low	Moderate	Low	Low	Low	Low	Low	Low	High	Low		High	

## Discussion

This systematic review aimed to discover the association of hormonal contraceptives with oral manifestations. Regarding the results, it is essential to highlight the presence of two high-risk articles [36, 37]. The low reliability obtained from the quality analysis justifies the decision to discard its data.

It has been reported that women who take oral contraceptives have a higher risk of experiencing alveolar osteitis after a dental extraction because estrogen causes a variation in coagulation and fibrinolytic factors, leading to a more significant dissolution of clots and hindering proper healing [3]. Parthasarathi et al. [44] examined a group of individuals who underwent extractions. They reported that none of the patients taking contraceptives subsequently developed alveolar osteitis. However, meta-analyses, including by Bienek et al., [45] determined that the use of oral contraceptives almost doubles the risk of developing alveolar osteitis, while Tang et al. [46] indicated that a relationship may exist between hormonal dosage and the incidence of alveolar osteitis.

Aminzadeh et al. [35] attempted to link the use of combined oral contraceptives with oral candidiasis. Estrogen has been reported to enhance the growth and adhesion of the *Candida* species to vaginal epithelial cells [47], and Aminzadeh et al. [35] investigated the potential colonization of these species in the oral cavity of women who take oral contraceptives containing estrogen. They concluded that oral contraceptives may increase the colonization of *C. albicans* and *C. krusei*, but their use did not determine the development of the disease in these women. Furthermore, they recommended conducting further research to link the use of oral contraceptives to the adherence of the *Candida* species in the oral epithelium, similar to what other authors, including Gonçalves et al. [48], have recommended regarding vaginal candidiasis.

Steroid hormones can indirectly lead to changes in periodontal tissue [13, 49], and the presence of estrogen and progesterone receptors has been demonstrated in the gingivae. Estrogen receptors are present in periosteal fibroblasts, fibroblasts scattered in the lamina propria, and fibroblasts and osteoblasts of the periodontal ligament [3, 13]. These receptors bind to specific hormones, which accumulate and are retained within the tissues and can bring about changes in gingival response [13]. Mullally et al. [42] reported that women using combined oral contraceptives had poorer periodontal health. Studies, including the research conducted by Domingues et al. [50], indicate that combined oral contraceptives can impact the periodontal condition in women, potentially leading to heightened gingival inflammation.Additionally, Mullally et al. [42] determined that, among women with aggressive forms of periodontal disease such as generalized aggressive periodontitis, the current or prior use of combined oral contraceptives was common. Therefore, they reported that medication, even in formulations with less estrogen, promoted increased periodontal destruction in patients susceptible to the disease. However, Taichman and Eklund [40] concluded that low-dose and high-dose combined oral contraceptives were not associated with the increased occurrence of gingivitis or periodontitis but were unable to determine a protective effect from them. Their study presented a representative sample of the population, as they obtained information through national health and nutrition examination surveys (NHANES). The self-selection of data to make the surveys comparable and the use of different methods to evaluate the presence or absence of periodontal disease may have influenced the study’s outcome. Perhaps contraceptives might not have adverse effects on most women’s periodontal health, but they could be considered a risk for those susceptible to aggressive forms of periodontal disease [42].

Brusca et al. [32] supported Mullaly et al.‘s [42] theory that female users of combined oral contraceptives had worse periodontal health due to higher and significant levels of severe periodontitis. Moreover, Brusca et al. [32] determined that these patients presented a higher number of specific periodontal pathogens in periodontal pockets, including *Porphyromonas gingivalis*, *Prevotella intermedia*, *Aggregatibacter actinomycetemcomitans*, and a wide variety of *Candida* species. Specifically, *C. albicans, C. parapsilosis, C. krusei, C. tropicalis*, and *C. glabrata* were the species capable of surviving after 3 years of hormonal therapy, while *C. dubliniensis* was not isolated in the periodontal pockets. A more recent study by Arumugam et al. [51] confirmed the influence of oral or injectable contraceptives on the occurrence of *Candida* species in periodontal pockets. However, both studies reported that the association between *Candida* and periodontitis was controversial.

Haerian-Ardakani et al. [33] studied a group consisting of women taking low-dose combined oral contraceptives for at least 2 years. Their findings were consistent with those of Mullally et al. [42] in that these women showed more bleeding and the presence of gingivitis.

Wu et al. [38] and Smadi and Zakaryia [34] also determined that the use of oral contraceptives exacerbated gingival inflammation and the development of gingivitis. These results are consistent with previous studies such as the one by Tilakaratne et al. [52], which argue that hormonal contraceptives are associated with a higher prevalence of gingivitis.

Furthermore, Smadi and Zakaryia [34] indicated that androgens played a relevant role in maintaining bone mass and could suppress osteoclastic functions or the synthesis of prostaglandins and interleukin 6 (IL-6) during an inflammatory process. They also stated that recently introduced oral contraceptives, in addition to lower estrogen doses, exhibited changes in the progesterone component to induce fewer androgenic side effects. The fact that the effects on the periodontium remained evident led them to conclude that gingival disease was exacerbated by the use of new generations of combined oral contraceptives, which lacked the potential androgenic protective effect. This study highlighted the need for research that examines the effect of each type of progesterone on oral health, including those in contraception methods using different administration routes.

Prachi et al. [39], based on the community periodontal index and the index of loss of attachment, determined that women taking oral contraceptives had worse periodontal and gingival health. Their results were consistent with those of Mullally et al. [42]. Furthermore, Prachi et al. [39] reported a significant association between these indices and the duration of contraceptive therapy. A review by Ali et al. [53] determined that changes in the periodontium appeared after a few months and gradually increased with the duration of therapy.

Taichman et al. [41] investigated the relationship between periodontal diseases and the injectable method of medroxyprogesterone acetate (DMPA). They observed a significant association between the current use of DMPA and gingivitis and a modest association with periodontitis. The study by Bagheri et al. also supports that the use of DMPA has effects on the periodontal health of patients [54].

However, in Taichman et al.‘s study, [41] it was puzzling that female smokers using DMPA had a lower likelihood of periodontal disease. This could lead to speculation that both factors do not synergistically increase the risk of periodontitis, but further studies are needed to clarify this finding. Similar to Taichman and Eklund [40], this data is cross-sectional and based on NHANES surveys. Authors like Eke et al. [55] criticize that the periodontal examinations they use underestimate the prevalence of periodontitis, leading to inaccurate results in disease classification.

Finally, Bostanci et al. [43] studied the influence of hormonal fluctuations on the dysbiosis or imbalance of the salivary microbiome. The study reported that hormonal contraceptives were not associated with significant changes in the salivary microbiome. These findings were consistent with those of Krog et al., [56] who reported that hormonal contraceptives used by healthy young women did not have a significant relationship with the composition of the salivary microbiome.

Limitations of the study included that articles that did not include the terms used in the search strategy, whether related to hormonal contraception or the oral cavity, were not evaluated. In addition, the search, to select articles effectively, was limited to the past 20 years due to low search result figures. To the authors’ knowledge, this was the first systematic review that evaluated the different oral manifestations that may appear in women using any form of hormonal contraception. New research that avoids variability in study execution and considers potential modifying factors such as the type of hormonal compound, hormonal dosage, therapy duration, or tobacco use is essential to evaluate the oral manifestations produced by various types of hormonal contraceptives. This approach will enable precise comparisons to establish robust conclusions about the topic. By investigating whether such hormones increase the risk of oral manifestations, we can present comparative data between women undergoing therapy and those not in treatment. Therefore, establishing a single experimental protocol is necessary to facilitate the interpretation of the data obtained. This will allow us to extrapolate our results to the general population It is crucial to note that while a meta-analysis would ideally offer a quantitative comparison of the effects of hormonal contraceptive methods on oral manifestations, conducting such an analysis is not feasible within the current scope of our research. The limited availability of specific studies and the heterogeneity of the reported data prevent us from performing a meta-analysis with the necessary statistical rigor. This limitation underscores the need for further research in this area to accumulate a more extensive and homogeneous data set, enabling future meta-analytical evaluations.

## Conclusions

Based on the results, we determined that hormonal contraceptives may increase the risk of alveolar osteitis after tooth extraction and promote the presence of the *Candida* species in the oral cavity. However, no evidence of their association with the development of oral candidiasis was found. Additionally, hormonal contraceptives affect the periodontium, including increased gingival inflammation, worsened development of periodontitis in susceptible patients, and a higher presence of specific periodontopathogens in periodontal pockets. Hormonal contraceptives, however, are not associated with significant changes in the salivary microbiome.
